# Effects of surgical approach and downstaging in esophageal adenocarcinoma patients treated with neoadjuvant chemotherapy: a 2010–2020 National Cancer Database (NCDB) study

**DOI:** 10.1007/s00464-024-11495-2

**Published:** 2025-01-23

**Authors:** Eduardo A. Canto, Matthew Reilly, Alexander Hall, Ryan W. Walters, Kalyana C. Nandipati

**Affiliations:** 1 School of Medicine, Creighton University, Omaha, USA; 2 Department of Surgery, Creighton University, Omaha, USA; 3 Department of Clinical Research and Public Health, Creighton University, Omaha, USA; 4 Wake Forest School of Medicine, Charlotte Campus, Winston-Salem, NC USA; 5 Department of Surgery, School of Medicine, Creighton University, 7710 Mercy Road, Education Building, Suite 501, Omaha, NE 68124 USA

**Keywords:** Adenocarcinoma, Esophagus, Neoadjuvant therapy, Downstaging

## Abstract

**Background:**

Neoadjuvant Chemoradiation (nCRT) has been shown to improve survival in patients with Esophageal Adenocarcinoma (EAC). The objective of this study is to assess the patient characteristics associated with tumor downstaging in a large national database. Additionally, we evaluated surgical approach and change in clinical versus pathological staging as predictors of patient survival.

**Methods:**

Using the 2010–2020 National Cancer Database, we identified 6,400 patients with clinical stage 1B to 4A EAC who received nCRT and underwent esophagectomy. Multivariable logistic models were estimated to evaluate odds of downstaging, and complete downstaging. Multivariable marginal Cox proportional-hazard models were estimated to evaluate all-cause mortality hazard.

**Results:**

3285 (51%) patients downstaged (of which 292 [5% of total] completely downstaged), 2430 (38%) had no change in stage, and 685 (11%) progressed. Generally, higher covariate values such as Clinical T, Clinical N, age, and Charlson-Deyo score were associated with higher odds of downstaging and lower odds of complete downstaging. Downstaging was associated with 31% lower risk of death compared to progression (p < .001) and 17% lower risk of death compared to no change (p < .001). Regarding surgical approach, when compared with open esophagectomy (OE), robotic-assisted minimally invasive esophagectomy (RAMIE) was associated with 17% lower adjusted risk of death (p = .002) while minimally invasive esophagectomy (MIE) was associated with a 10% decrease in adjusted risk of death (p = .009).

**Conclusion:**

In patients with EAC who underwent nCRT, pathological downstaging was associated with increased survival compared to no change or progression. Additionally, RAMIE and MIE were associated with lower risk of death compared to OE.

**Graphical Abstract:**

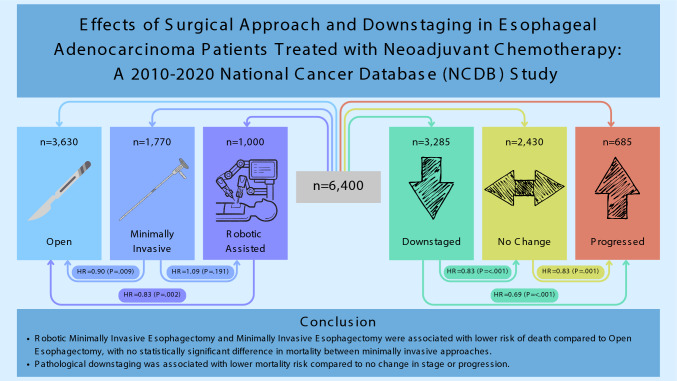

Esophageal cancer (EC) is the sixth leading cause of cancer-related deaths worldwide [1]. In the United States in 2023, EC accounted for 1.1% of all cancer diagnoses and 2.6% of all cancer deaths [2] with esophageal adenocarcinoma (EAC) accounting for 64% of all esophageal cancers [3]. A diagnosis of EAC at a localized stage results in an almost 10 times greater 5-year survival compared to diagnosis with distant metastasis [4]. However, because more than two thirds of newly diagnosed patients have regional and metastatic disease on presentation [4], esophagectomy alone will not suffice. According to the American Society of Clinical Oncology (ASCO), there are two modalities of treatment that should be offered to patients with locally advanced EAC (stages IIb-IIIc). These include neoadjuvant chemotherapy with docetaxel, oxaliplatin, leucovorin, and fluorouracil (FLOT) or neoadjuvant chemoradiotherapy (nCRT) followed by esophagectomy [5]. Similarly, patients with EAC that received nCRT had a 25% decrease in all-cause mortality [6]. Though data comparing the rates of stage-specific downstaging for EAC and related survival outcomes are scarce. The survival advantages seen in patients treated with nCRT may be explained by nCRT’s role in eliminating micro-metastases and downstaging at the primary tumor site [7]. Previous studies utilizing small cohorts of patients showed that downstaging was associated with longer survival compared to those without downstaging at the time of surgery [8]. Additional studies have also shown that nCRT’s effect on lymph node regression may play a stronger role in EAC prognosis than nCRT’s effect on the primary tumor itself [9].

In addition to nCRT, the approach used for esophagectomy may also be associated with improved patient outcomes. There are three surgical approaches to esophagectomy: open (OE), minimally invasive (MIE), and robotic-assisted minimally invasive (RAMIE). RAMIE has been shown to provide superior oncologic resection margins without increasing risk of mortality when compared to OE and other non-robotic MIE techniques [10]. Furthermore, RAMIE and MIE have both been associated with reduced postoperative morbidity and shorter hospital stays; however, their effect on EAC long-term mortality has not been compared with OE approaches [11, 12].

The primary aim of this study is to determine downstaging in patients treated with nCRT and examine characteristics associated with partial and complete downstaging. Additionally, we aim to understand how surgical approach and change in post-nCRT staging (downstaging, no change, or progression) are related to survival outcomes in this patient cohort.

## Methods

### Data source

Data for this project were abstracted using the 2020 esophagus participant user files (PUF) from the National Cancer Database (NCDB) [13]. NCDB data are deidentified, HIPAA-compliant data sets, comprising cases submitted to the Commission on Cancer (CoC) of the American College of Surgeons and the American Cancer Society. Within the United States, NCDB PUF’s capture approximately 72% of newly diagnosed cases of cancer from more than 1,500 commission-accredited cancer programs [14].

### Ethical considerations

This study was reviewed and acknowledged as not human subjects research by the Creighton University Institutional Review Board. The review was conducted under InfoEd record number 2004110.

### Patient cohort

We identified adult patients with clinical stage 1B to 4A EAC who received nCRT and underwent esophagectomy. Diagnosis of adenocarcinoma was defined using histology and behavior codes (Histology 8141/3, 8143, 8147, 8255, 8260/3, 8261–8263, 8480/3, 8481/3, 8570–8573, and 8574/2, with primary site of lower two-thirds esophagus (C15.1, C15.2, C15.4, C15.5) defined using ICD-10 diagnosis codes, who received neoadjuvant chemoradiation defined separately as chemotherapy (single-agent, multiagent; type and number of agents not documented) and radiation therapy (before or before and after surgery). Clinical staging was identified using 7th and 8th edition AJCC clinical stage group, and due to discrepancies between editions (7th edition frequencies show patients in 1B, 2, 2A, 2B, 3, 3A, 3B, 3C; 8th edition frequencies show patients in 2A, 2B, 3, 4A), collapsed into categories 1B, 2, 3, 4A. Similarly, pathologic staging was identified using 7th and 8th edition AJCC pathologic stage group (7th edition frequencies show patients in 0, 1, 1A, 1B, 2, 2A, 2B, 3, 3A, 3B, 3C, 4; 8th edition frequencies show patients in 0, 1A, 1B, 1C, 2A, 2B, 3A, 3B, 4A, 4B), and collapsed into categories 0, 1, 2, 3, 4. The study range was selected from 2010 to 2020 due to 2010 being the first year which a surgical approach variable was available in the NCDB. Patients were excluded if they were missing clinical or pathological staging information.

### Definition of downstaging

Downstaging was identified by comparing the collapsed 7th and 8th edition AJCC pathological stage to pre-nCRT clinical stage—consistent with similar recent work [15]. Response to nCRT was defined for each patient as downstaged (pathological < clinical), no change (pathological = clinical), or progression (pathological > clinical). Complete downstaging was defined as pathologic stage 0 (i.e., pT0N0).

### Statistical analysis

Overall patient descriptive statistics are presented as median and interquartile range for continuous variables and frequency and percent for categorical variables. Multivariable logistic models were estimated to evaluate odds of downstaging, and complete downstaging, whereas multivariable marginal Cox proportional-hazard models were estimated for all-cause mortality. Additionally, for each outcome, two-way interactions between clinical stage and each covariate were separately estimated to evaluate whether the effect of each covariate was statistically similar across clinical stages. Downstaging covariates included clinical T (1, 2, ≥ 3) and N (0, ≥ 1) category, age at diagnosis, biological sex, race (White vs Non-White), ethnicity (Hispanic vs Non-Hispanic), Charlson-Deyo score (0, 1, ≥ 2), as well as type of chemotherapy delivered (single agent vs. multiagent). Mortality covariates included age at diagnosis, biological sex, race (White vs Non-White), presence of regional lymph nodes (0, ≥ 1), Charlson-Deyo score (0, 1, ≥ 2), and procedure type (total esophagectomy – not otherwise specified (NOS), esophagectomy w/ gastrectomy NOS, esophagectomy NOS, esophagectomy with partial gastrectomy).

## Results

### Patient characteristics

In total, 6400 adult patients with clinical stage 1B to 4A EAC who received nCRT and underwent esophagectomy were identified in the NCDB. Our patient sample primarily consisted of older, White males, with a clinical stage 2 or 3 EAC with open surgical approach (Table 1).

**Table 1:** Patient characteristics

N = 6400	Frequency (%)
Male	5703 (89%)
White	6162 (96%)
Hispanic	164 (3%)
Adenocarcinoma, NOS	6226 (97%)
Lower third of esophagus	6105 (95%)
Multiagent chemo as first course	5805 (91%)
Systemic therapy before surgery (compared with both before and after)	5792 (91%)
Initial clinical stage	
1B	282 (4%)
2	1971 (31%)
3	3816 (60%)
4A	331 (5%)
Pathological stage	
0	292 (5%)
1	1900 (30%)
2	1898 (30%)
3	2127 (33%)
4	183 (3%)
Surgical approach	
Robotic assisted	1000 (16%)
Minimally invasive	1770 (28%)
Surgical procedure	
Total esophagectomy, NOS	858 (13%)
Esophagectomy with gastrectomy, NOS	1511 (24%)
Esophagectomy with partial gastrectomy	3783 (59%)
Esophagectomy, NOS	248 (4%)
	Median (IQR)
Age	63 (57, 69)

### Downstaging

When comparing clinical stage at presentation to pathological stage at surgery, 3285 patients (51%) downstaged, 2430 (38%) had no change in stage, and 685 (11%) experienced progression. Evaluating the overall adjusted model, higher covariate values such as Clinical T, Clinical N, age, and Charlson-Deyo score were associated with higher odds of downstaging (Table 2). Statistically significant predictors included Charlson-Deyo score; compared to a score of 0, a score of 1 resulted in 14% higher odds of any downstaging (OR: 1.14, 95% CI: 1.00–1.29, p = 0.042). Age was also a statistically significant predictor; every 10-year increase in age was associated with an estimated 8% increase in odds of downstaging (OR: 1.08, 95% CI:1.02–1.14, p = 0.006). Further, clinical N of ≥ 1 compared with 0 was associated with 108% higher odds of any downstaging (OR: 2.08, 95% CI: 1.87–2.31, p < 0.001), and clinical T of ≥ 3 compared with 1 was associated with an estimated, though non-significant, 36% higher odds of any downstaging (OR: 1.36, 95% CI: 0.99–1.87, p = 0.055). Concerning interactions between covariates and clinical stage, a statistically significant two-way interaction was observed for both T clinical and N clinical, suggesting that the overall model odds ratio may not apply similarly to each stage; however, this interaction appears to be a matter of magnitude rather than direction. For example, among patients with clinical stage 2, a T clinical score of ≥ 3 was associated with 57% lower odds of downstaging than a T clinical score of 2 (OR: 0.43, 95% CI: 0.34–0.55, p < 0.001), whereas among patients with clinical stage 3, a T clinical score of ≥ 3 was associated with 26% lower odds of downstaging than a T clinical score of 2 (OR: 0.74, 95% CI: 0.57, 0.96, p = 0.024).

**Table 2:** Odds ratios for downstaging and complete downstaging

	Downstaging		Interaction	Complete downstaging		Interaction
	aOR (95% CI)	*p*	*p*	aOR (95% CI)	*p*	*p*
Clinical T			**.025**			.729
2 vs. 1	1.21 (0.87, 1.69)	.257		1.05 (0.56, 1.97)	.886	
≥ 3 vs. 1	1.36 (0.99, 1.87)	.055		**0.53** (0.29, 0.98)	.042	
≥ 3 vs. 2	1.13 (0.99, 1.28)	.069		**0.51** (0.39, 0.66)	< .001	
Clinical N			** < .001**			.442
≥ 1 vs. 0	**2.08** (1.87, 2.31)	< .001		0.95 (0.84, 1.09)	.710	
Age (per 10 years)	**1.08** (1.02, 1.14)	.006	.259	0.96 (0.84, 1.09)	.504	.999
Male vs. female	1.11 (0.94, 1.31)	.229	.534	0.79 (0.52, 1.21)	.278	.636
White vs. non-white	1.07 (0.80, 1.42)	.652	.897	–		–
Hispanic vs. non-hispanic	1.13 (0.82, 1.55)	.473	.699	1.07 (0.52, 2.23)	.8481	.168
Charlson-Deyo			.344			.511
≥ 2 vs. 0	1.09 (0.91, 1.31)	.330		**0.49** (0.28, 0.86)	.012	
1 vs. 0	**1.14** (1.00, 1.29)	.042		0.99 (0.74, 1.33)	.964	
Chemotherapy type			.479			.975
Single-agent vs. multiagent	1.01 (0.71, 1.42)	.970		**1.93** (1.05, 3.56)	.035	

Interaction P shows the p value for the 2 × 2 interaction between each covariate and Clinical Stage Group; for covariates with statistically significant interactions, the associated overall model odds ratio may not apply similarly to each stage

Bold indicates statistical < .05

*aOR* model adjusted odds ratio, *CI* Confidence Interval

### Complete downstaging

Of all 6400 patients, 292 (5%) experienced complete downstaging, of whom 34 (12%) came from clinical stage 1B (4% of study population), 130 (45%) from stage 2 (31% of study population), 128 (44%) from stage 3 (60% of study population) and no patients with clinical stage 4A experienced complete downstaging (5% of study population). Evaluating the overall model adjusting for all included covariates, higher covariate values were generally associated with lower estimated odds of complete downstaging (Table 2). For example, patients with clinical T of ≥ 3 showed a 47% and 49% decrease in the odds of complete downstaging when compared with a Clinical T of 1 or 2, respectively (OR: 0.53, 95% CI: 0.29–0.98, p = 0.042; OR: 0.51, 95% CI: 0.39–0.66, p < 0.001, respectively). Further, compared with a Charlson-Deyo score of 0, a Charlson-Deyo of ≥ 2 was associated with a 51% decrease in the odds of complete downstaging (OR: 0.49, 95% CI: 0.28–0.86, p = 0.012). No interactions for complete downstaging models were statistically significant, suggesting that overall model odds ratios apply similarly to each stage.

### Mortality

**Fig. 1:**
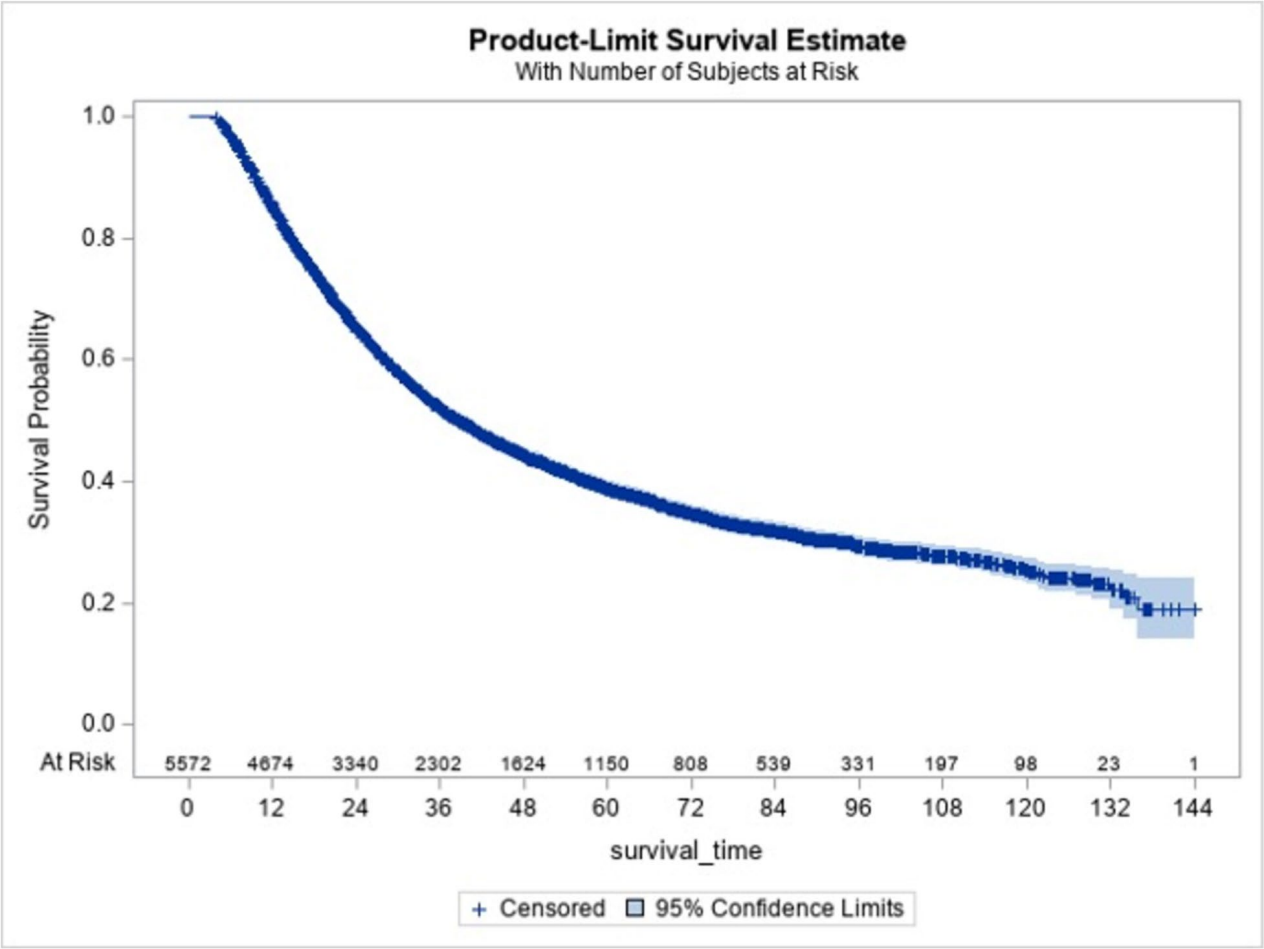
Product-limit survival estimate

Patients were followed for a median of 30 months (IQR: 16–54 months), with 3271 (59%) suffering all-cause mortality and 2301 (41%) being censored. Figure 1 shows the overall unstratified Kaplan–Meier survival curve with a median time-to-death of 38.5 months (95% CI: 36.6–40.71). Table 3 displays the adjusted hazard ratio for covariates included in the Cox model. Binary downstaging and surgical approach were both associated with adjusted risk of death (Table 3). Specifically, downstaging was associated with 31% lower risk of death compared to progression (HR: 0.69, 95% CI: 0.60–0.77, p < 0.001) and 17% lower risk of death compared to no change (HR: 0.83, 95% CI: 0.76–0.90, p < 0.001). Patients with no change averaged 17% lower risk of death compared to patients whose EAC progressed (HR: 0.83, 95% CI: 0.75–0.92, p < 0.001). Regarding surgical approach, when compared with OE, RAMIE was associated with 17% lower adjusted risk of death (HR: 0.83, 95% CI: 0.73–0.94, p = 0.002) and MIE was associated with a 10% decrease in adjusted risk of death (HR: 0.90, 95% CI: 0.83–0.97, p = 0.009). No statistically significant difference in risk of death was observed when comparing MIE to RAMIE (HR: 1.09, 95% CI: 0.96–1.23, p = 0.191). Additionally, compared with patients who had 0 regional nodes, those who were node positive showed a 67% higher risk of death (HR: 1.67, 95% CI: 1.52–1.81, p < 0.001). Additionally, age, Charlson-Deyo, and female compared to male were statistically significant predictors of risk of death (Table 3). Procedure type was not a statistically significant predictor of risk of death (Table 3). The interaction effect for patients identified as White compared with those identified as belonging to other Non-White racial categories was statistically significant (*p* = 0.027). No other statistically significant two-way interactions were observed between clinical stage and any covariate indicating that HRs reported below apply similarly to each stage.

**Table 3:** Risk of death

Variable	HR	95% CI	*P*	*Interaction P*
Approach				.671
Minimally invasive vs. open	**0.90**	0.83–0.97	.009	
Minimally invasive vs. robotic	1.09	0.96–1.23	.191	
Robotic vs. open	**0.83**	0.73–0.94	.002	
Staging change				.117
Downstage vs. progression	**0.69**	0.61–0.77	< .001	
Downstage vs. no change	**0.83**	0.76–0.90	< .001	
No change vs. progression	**0.83**	0.75–0.92	.001	
Regional nodes vs. no regional nodes	**1.67**	1.52–1.82	< .001	.156
Age (10-year change)	**1.18**	1.13–1.30	< .001	.484
Charlson-Deyo				.942
3	**1.39**	1.16–1.67	< .001	
2	**1.32**	1.16–1.51	< .001	
1	**1.22**	1.13–1.30	< .001	
0	REF			
Female vs. male	**0.86**	0.76–0.97	.016	.190
White vs. non-white	0.87	0.71–1.07	.193	**.027**
Procedure				.929
Total esophagectomy NOS	1.05	0.95–1.17	.345	
Esophagectomy w/ gastrectomy NOS	1.11	0.99–1.22	.051	
Esophagectomy NOS	0.93	0.76–1.13	.447	
Esophagectomy w/ partial gastrectomy	REF			

Bold indicates statistical < .05

*CI* confidence interval

## Discussion

Our results showed that in esophageal adenocarcinoma (EAC) patients treated with neoadjuvant chemoradiotherapy (nCRT), downstaging status and surgical approach (MIE and RAMIE) were significant predictors of all-cause mortality. The role of neoadjuvant treatment, especially neoadjuvant chemoradiation, has been well-established in locally advanced esophageal adenocarcinoma [5]. nCRT has shown to improve margin-free resection and overall survival, most likely through downstaging. In our study, downstaging was associated with a 31% decreased risk of all-cause mortality compared to no change or progression. We also noted that no change status was associated with a 17% lower risk of mortality compared to progression. Our results support the previously published literature indicating that nCRT and downstaging, which is a surrogate marker for response, are associated with better overall survival [5, 15, 16]. The role of nCRT in esophageal adenocarcinoma has still been debated given the impact on cardiopulmonary function. However, given the overall improvements in survival, our study highlights the importance of nCRT in the treatment regimen of EAC and should be offered to all patients who are qualified according to the NCCN guidelines and able to tolerate.

The overall survival benefit reported from the downstaging makes a compelling case for nCRT [16]. Our study showed that more than half of the EAC patients that received nCRT achieved pathological downstaging, which was similar to the previous reports [5, 15–17]. Although individual clinical variables have been studied as predictors of downstaging with nCRT, the association between clinical stage and odds of downstaging has not been evaluated in EAC. Of the factors that impact the results, the accuracy of clinical stage and its concordance with pathological stage has been controversial. The concordance rates have been noted to be variable between 65.2 and 98.8% [18]. The concordance rates have been inversely related to stage as evidenced by the higher the clinical stage, the lower the concordance, and similarly, in the lower stage there was higher concordance [18]. The variability makes downstaging accuracy questionable, especially when lymph nodes (LN) are involved. Our results showed that clinical LN positivity was associated with higher odds of responding to nCRT with pathological downstaging but T ≥ 3 didn’t show a similar response. This highlights the importance of clinical staging especially LNs involvement which can help in guiding patients towards nCRT.

Of the patients who achieved downstaging, a small portion (5%) achieved complete downstaging. However, the rate of complete pathological downstaging in this study was lower compared to the previous published report that showed a 23% complete response in patients with adenocarcinoma [16]. The possible reasons behind the low complete response rates in our study includes inclusion of higher stage patients in the neoadjuvant group and heterogeneity in the chemo regimen (single vs multiple). On the other hand, the majority of literature reports [16, 17] have a small sample size compared to our study. Further sub-analysis of completely downstaged patients also showed an interesting finding: patients with T2 completely downstaged at twice the rate compared to the patients with T3. Patients with T3 had higher tumor burden which might be the main reason behind the lower rate of complete response. There was no such difference noted with N+ status. Overall, our study highlights the importance of advocating for neoadjuvant chemoradiation at T2 tumors. Our results also showed that not only tumor burden, but also patients with higher Charlson-Deyo score were associated with lower odds of complete downstaging. This highlights the role of the comorbidity index, which not only has an impact on the surgical outcomes, but also in the response to nCRT. Interestingly, patients who received single-agent chemo were almost twice as likely to achieve complete downstaging. It is difficult to draw conclusions from this finding since the vast majority (91%) of the patients received multiagent chemo which has been the standard of care.

Current research shows that nCRT leads to tumor downstaging and improves survival in patients with EAC [15–17]. Additionally, downstaging has been considered as an independent predictor of significant improved overall survival [8]. Our results corroborated previous literature suggesting that nCRT has been an independent risk factor for overall survival. Since our study also has significant numbers with complete pathological response, we were able to show complete responders have significantly better survival odds compared to the other population. Previous research reported that there is an association between the degree of downstaging and survival and pathological complete resection (R0) [15, 17]. Our study demonstrates that even patients with no change in stage with nCRT resulted in a significant improvement in survival compared to progression after nCRT. Clinically, this suggests that patients with or without significant comorbidity index and those who can tolerate nCRT, should be considered for this modality as recommended in the current guidelines [19]. Currently, subpopulations who progress on nCRT are difficult to predict from the tumor characteristics. More research is needed to identify this subset of the population. This will help us to subcategorize these population subsets and employ targeted treatment options.

Studies show that increased lymph node removal in EAC patients treated with esophagectomy alone is associated with decreased mortality [20]. The extent to which this second set of findings apply to patients treated with nCRT is unknown. In our study, the presence of positive regional nodes was associated with higher mortality. Overall number of LN’s removed in nCRT patients and their impact on overall survival has been controversial. It is evident that patients with positive LNs following nCRT are associated with significantly poorer survival than overall number of LNs. The number of LNs removed can be associated with more positive yield but this notion hasn’t been well established. It is essential that LN dissection is important in prognosis, but it doesn’t impact adjuvant treatment as any residual cancer after nCRT will need adjuvant treatment. It is unlikely that patients will have pathological positive LNs without residual primary tumor.

Finally, in our study, MIE and RAMIE were associated with decreased mortality compared to OE. Our study likely had selection bias in terms of surgical approach used. Sicker patients could have been overrepresented in the OE group. Previous research shows that in patients with EAC who did not undergo nCRT, OE was associated with higher mortality than MIE [21]. However, the disease-free survival at 3 years was similar between both groups in that study. Given that previous research shows disease-free survival and lymph node yield to be similar between MIE and OE, MIE should be the desirable surgical approach in eligible patients with EAC due to its lower mortality rate. RAMIE has been reported to have improved overall survival and increased lymph node yield compared to MIE and OE in previous reported literature [21, 22]. In this study, RAMIE had better survival compared to MIE; however, this was not statistically significant, likely due to a lack of sufficient sample size. Studies addressing this question are currently ongoing and will provide concrete data when prospective randomized controlled trials comparing RAMIE and MIE are published.

## Limitations

The NCDB is retrospective with the inability to collect additional data, and of relevance to this study, there could potentially be data entry errors with TNM and clinical staging. Further, the NCDB only enables the analysis of all-cause mortality. Additionally, the American Joint Committee on Cancer published the 7th and 8th edition for EAC staging between 2010 and 2020, and there are some nuances to clinical staging and the stage (for this reason we group sub-stages together e.g. 2A and 2B as stage 2). As noted in the discussion section, there is variability in clinical and pathological stage concordance. Additionally, the NCDB does not provide information on the rationale for each patient’s surgical approach nor chemotherapy regimen provided, which limits our ability to assess to what degree selection bias influenced surgical decision-making or chemotherapy choice.

## Conclusions

In patients with esophageal adenocarcinoma who underwent neoadjuvant chemoradiation, pathological downstaging was associated with lower mortality risk when compared to the patients who had no change in stage or progressed. RAMIE and MIE were associated with lower risk of death compared to OE, with no statistically significant difference in mortality between RAMIE and MIE.
